# Substantia Nigra Hyperechogenicity Reflects the Progression of Dopaminergic Neurodegeneration in 6-OHDA Rat Model of Parkinson’s Disease

**DOI:** 10.3389/fncel.2020.00216

**Published:** 2020-08-04

**Authors:** Siyan Zhang, Kai Tao, Jia Wang, Yunyou Duan, Bao Wang, Xi Liu

**Affiliations:** ^1^Department of Ultrasound, Second Affiliated Hospital of Air Force Medical University, Xi’an, China; ^2^Department of Neurosurgery, Second Affiliated Hospital of Air Force Medical University, Xi’an, China; ^3^Department of Ultrasound, Air Force Medical Center, Air Force Medical University, Beijing, China

**Keywords:** Parkinson’s disease, transcranial sonography, substantia nigra hyperechogenicity, dopaminergic neuron, microglia activation

## Abstract

Parkinson’s disease (PD) is the second most common neurodegenerative disease, and there is still no effective way to stop its progress. Therefore, early detection is crucial for the prevention and the treatment of Parkinson’s disease. The current diagnosis of Parkinson’s disease, however, mainly depends on the symptoms, so it is necessary to establish a reliable imaging modality for PD diagnosis and its progression monitoring. Other studies and our previous ones demonstrated that substantia nigra hyperechogenicity (SNH) was detected by transcranial sonography (TCS) in the ventral midbrain of PD patients, and SNH is regarded as a characteristic marker of PD. The present study aimed to explore whether SNH could serve as a reliable imaging modality to monitor the progression of dopaminergic neurodegeneration of PD. The results revealed that the size of SNH was positively related with the degree of dopaminergic neuron death in PD animal models. Furthermore, we revealed that microglia activation contributed to the SNH formation in substantia nigra (SN) in PD models. Taken together, this study suggests that SNH through TCS is a promising imaging modality to monitor the progression of dopaminergic neurodegeneration of PD.

## Introduction

Parkinson’s disease (PD) is the second most common neurodegenerative disease that is age-related and characterized by the loss of dopaminergic neurons and neuroinflammation in the substantia nigra (SN) (Olson et al., 2016). While the causes of PD remain unclear, multiple therapies have been developed, including levodopa supplementation therapy, stem cell and deep brain stimulation, etc. In addition, a large amount of neuroprotective drugs are under investigation, such as nitidine, a pentacyclic alkaloid isolated from traditional herbal medicine *Zanthoxylum nitidum* (Roxb.) DC (Yuan et al., 2015), which could promote dopaminergic neuron survival by suppressing neuroinflammation (Wang et al., 2012, 2016; Yuan et al., 2015). However, all the abovementioned methods fail to stop the progression of dopaminergic neurodegeneration. Thus, early detection is crucial for the prevention and the treatment of Parkinson’s disease. The current diagnosis of Parkinson’s disease mainly depends on the symptoms, so it is necessary to establish a reliable imaging modality for PD diagnosis and its progression monitoring.

Other studies and our previous ones have found that substantia nigra hyperechogenicity (SNH) by transcranial sonography (TCS) was detected in the SN of PD patients, and this could differentiate PD patients from those with atypical parkinsonian syndromes (Becker et al., 1995; Behnke et al., 2005; Walter et al., 2005; Uwe et al., 2006; Okawa et al., 2007). More importantly, SNH could be detected in the SN (SNH) at the very early stages in PD patients (Berg et al., 2005; Berg, 2007; Gaenslen et al., 2008). A recent study showed that SNH was also correlated with the risk scores, motor signs, and prediagnostic features of PD (Noyce et al., 2018). Thus, the abovementioned findings suggest that SNH is a promising way for PD early detection and diagnosis.

The SN was identified within the butterfly-shaped structure of the mesencephalic brainstem, scanning from both temporal bone windows (Berg et al., 2005). Another interesting question worth exploring is the mechanism underlying SNH formation. The current researches on SNH are still limited. The previous studies have shown that the formation of SNH is highly related to iron accumulation in the SN region (Berg et al., 1999; Zhu et al., 2017). Although iron accumulation in SN is relevant to the neurodegeneration process in PD, it remains inconvincible to draw a direct conclusion that iron accumulation contributes to SNH and rule out other possibilities.

In this study, by establishing a 6-hydroxydopamine (6-OHDA)-induced unilateral PD rat model, we aimed to explore the relationship between SNH and dopaminergic neurodegeneration degree and primarily investigated the mechanism underlying SNH formation, with the hypothesis that SNH could serve as a reliable imaging modality to monitor the degree of dopaminergic neurodegeneration of PD.

## Materials and Methods

### Reagents

The following reagents were used in this study: tyrosine hydroxylase (TH) antibody and ionized calcium binding adaptor molecule-1 (Iba-1) antibody from Abcam (Cambridge, United Kingdom), β-actin antibody, HRP-conjugated goat polyclonal anti-rabbit IgG antibody, and biotinylated secondary antibody from Santa Cruz Biotechnology, Inc. (Santa Cruz, CA, United States), LPS and 6-OHDA from Sigma-Aldrich (St. Louis, MO, United States), and nitidine (purity >98%, by high-performance liquid chromatography) from Selleck Biotechnology Co., Ltd. Nitidine was dissolved in dimethyl sulfoxide at a final concentration of 1 mg/ml.

### Animals

Adult male Sprague–Dawley rats (*n* = 30) were purchased from the Animal Center of the Fourth Military Medical University (Xi’an, China) and housed at five rats per cage at a constant room temperature under a 12:12-h light/dark cycle. The rats were allowed free access to food and water. Surgery was performed when the rats reached 250–300 g of body weight. The rats were randomly divided into three groups: two groups were stereotactically injected with 6-OHDA and the third group was sham operated. All animal studies were approved by the Institutional Animal Care and Use Committee (IACUC-20190603).

### Establishment of 6-OHDA PD Rat Model

The rats were anesthetized with sodium pentobarbital (saline formulated as 0.3% solution) (35–45 mg/kg, i.p. injection) and placed in a stereotactic device (Stoelting, United States). An incision was made along the midline to expose the skull, and a 2-mm hole was drilled to allow the injection of 6.8 μl of 6-OHDA (dissolved in 0.02% ascorbic acid) or sham injection. A 10-μl Hamilton syringe attached to a glass capillary (outer diameter of 60–80 μm) was used to deliver injections at the following coordinates (mm): −1.80 AP and −4.92 ML from the anterior iliac crest and −8.00 DV from the anterior iliac crest [according to Paxinos and Watson (1998)]. The duration of each injection was at least 1 min. After the injection, the cannula was left in place for 5 min and then slowly withdrawn. The incision was sutured and disinfected with iodine. After completely recovering from anesthesia, the animals were returned to their cages.

The 6-OHDA solution was kept on ice for a maximum of 2 h and was protected from light throughout the process. Ascorbic acid in 0.02% sterile saline was used for the sham injections.

### Transcranial Sonography Examination

At 15 days after 6-OHDA administration, the rats were anesthetized and the skulls were surgically exposed. In order to remove the bone piece, the dura mater was preserved, and the rats were scanned when they were alive. After the surgery, we used Vevo 2100 high-frequency ultrasound (FUJIFILM VisualSonics Inc., Canada) with 25-MHz transducer to scan the midbrain planes of rats. Vevo anesthesia system was used for the maintenance of anesthesia. After the examination, Vevo Lab 3.1.1 was used to take the measurements.

According to Berg, the area of hyperechogenic signals in the SN region was encircled and measured. Results are given as mean and standard deviation and range (Berg et al., 2005). In our research, the images were frozen and enlarged four times, and the outline of the SNH was drawn carefully. The size of the delineated hyperechogenicity was automatically calculated. Each rat was repeatedly measured from multiple sections to get the maximum area of SNH. The images without SNH were recorded as 0 cm^2^.

For the mean gray values of SN, considering that SN is a three-dimensional structure, we randomly took three circular areas which were not larger than the size of SN, measured their mean gray values, and then took the average.

### Western Blot Analysis

The tissue was homogenized using radioimmunoprecipitation assay buffer (Sigma-Aldrich) mixed with a protease inhibitor cocktail and a phosphatase inhibitor (Complete Mini, Roche, Germany). A Pierce^TM^ BCA Protein Assay Kit (Thermo Fisher Scientific, Waltham, MA, United States) was used to determine the protein concentration. Protein samples (10 μg per lane) were separated by 10 and 15% (v/v) discontinuous sodium dodecyl sulfate-polyacrylamide gel electrophoresis and transferred to polyvinylidene fluoride membranes (Roche Diagnostics, Mannheim, Germany). Five percent skim milk was diluted in 0.1% Tween 20/Tris-buffered saline (TBS-T), and the membranes were blocked for 1 h at room temperature. The membranes were incubated with the primary antibody overnight at 4°C and washed three times for 5 min each in TBS-T on the next day. Then, the membrane was incubated with a suitable horseradish peroxidase-conjugated secondary antibody for 2 h at room temperature. Visualization was performed using an enhanced chemiluminescence method under standard protocols (ECL Plus; Thermo Fisher Scientific, United States). The anti-β-actin signal was used as a loading control. ImageJ (National Institutes of Health, Bethesda, MD, United States) was used for optical density assessment. Data are presented as the ratio between the TH (or Iba-1) and β-actin signals; the control value was set to 1.

### Immunohistochemistry

After the sections were rehydrated, they were heated in Tris/EDTA buffer (pH 9.0) for 10 min and then incubated with phosphate-buffered saline (PBS) blocking solution containing 5% normal goat serum at 4°C overnight. The sections were then incubated with biotinylated secondary antibody for 1 h, followed by incubation with peroxidase-conjugated avidin–biotin complex (ABC kit, Vector Laboratories, United Kingdom). 3,3-Diaminobenzidine (DAKO, Germany) was used as a peroxidase substrate. Incubation with secondary antibodies alone showed the specificity of the results. Standard hematoxylin-stained sections were used to visualize the nuclei. The treated sections were visualized and digitized using a Leica DMI 6000B (Leica, Wetzlar, Germany) fluorescence microscope workstation.

### Double-Immunofluorescence

The sections were incubated overnight at 4°C with immunofluorescent antibodies against TH (1:200) and Iba-1 (1:200) to identify dopaminergic neurons and activated microglia, respectively. After washing with PBS three times for 5 min each, the sections were incubated with the respective secondary antibody [goat anti-rabbit (1:200; Sigma) as TRITC for TH and goat anti-mouse as Cy3 for Iba-1 (1:200; Sigma)] for 2 h at room temperature. Colocalization of the markers was confirmed by confocal laser microscopy (TCS-SP2; Leica, Heidelberg, Germany) and sequential scanning methods.

### Data Analysis

Statistical analyses, including one-way ANOVA, *t*-tests, linear regression, and correlation analysis, were performed using GraphPad Prism 5 software (GraphPad Software Inc., San Diego, CA, United States). A *P* value ≤ 0.05 was regarded as statistically significant.

### Data Availability Statement

The data used to support the findings of this study are included within the manuscript and also available from the corresponding author.

## Results

### SNH Is Stably Observed in 6-OHDA PD Rat Models

**FIGURE 1:**
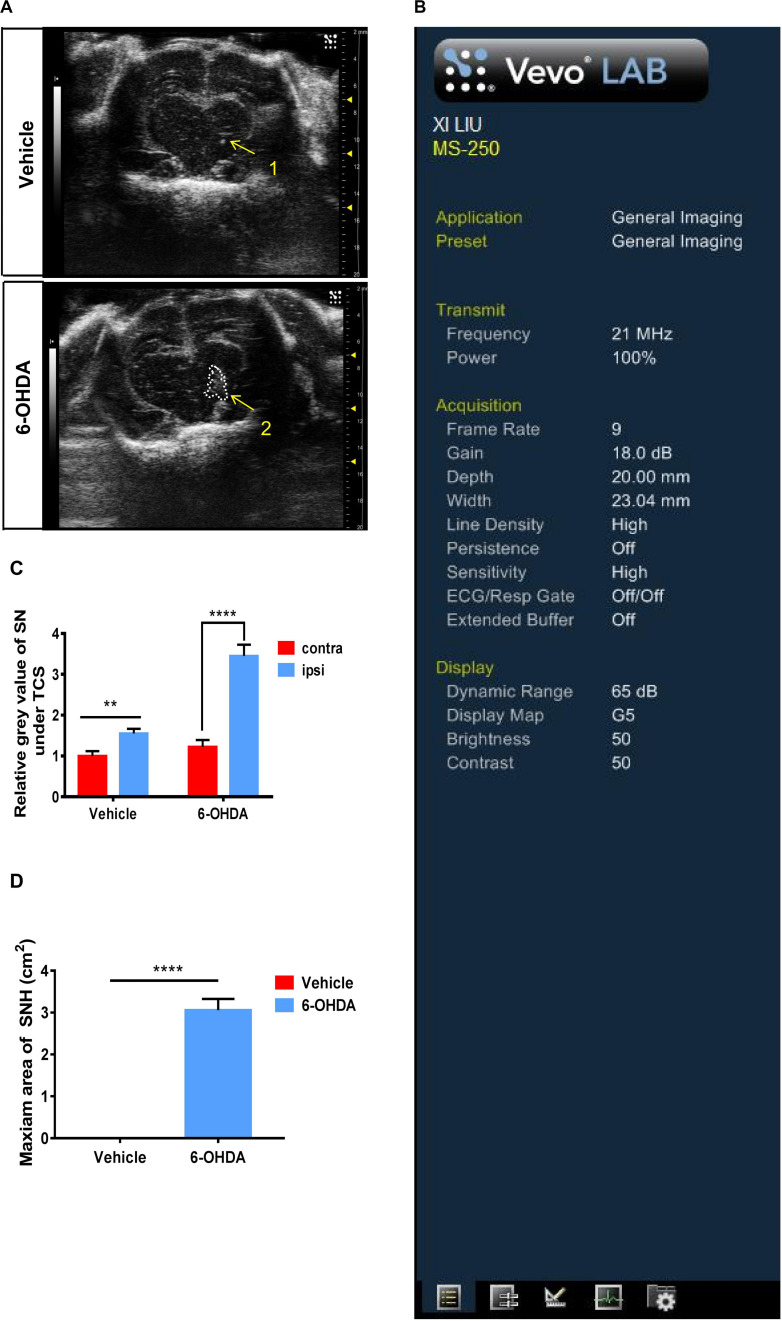
Transcranial sonography (TCS) images and qualification of substantia nigra hyperechogenicity (SNH) after unilateral stereotactic surgery. Male rats were injected with either 6-hydroxydopamine (6-OHDA) (*n* = 10) or vehicle (*n* = 10). At 15 days after surgery, TCS detection was performed. **(A)** Image of the midbrain plane showing a significant hyperechogenicity of 6-OHDA-lesioned rats (2), while only a scatter echo corresponding to the shape of the needle was observed in the vehicle group (1). Area 2 = 3.057 cm^2^. **(B)** General condition of TCS detection. **(C)** Statistical result of the mean gray values of substantia nigra (*t*-test). Vehicle group: *P* = 0.0015, *t* = 3.35, df = 50, *F* = 1.002. 6-OHDA group: *P* < 0.0001, *t* = 6.935, df = 52, *F* = 2.713. **(D)** Statistical result of the maximum SNH area (*t*-test). *P* < 0.0001, *t* = 11.44, df = 12. *Comparison between contralateral and ipsilateral from a single group. ns *P* > 0.05, **P < 0.01, ***P < 0.001, ****P < 0.0001.

We established unilateral PD rat models by a stereotactic injection of 6-OHDA into the SN areas. At 15 days after the 6-OHDA injection, typical hyperechogenicity in the SN was observed through TCS (Figure 1A). The ultrasonic detection parameters were also presented (Figure 1B). We digitized echogenicity imaging by measuring the SNH area and the mean gray values of SN, and the histograms showed a significant decrease in the mean gray value of the SN areas (Figures 1C,D). Immunofluorescence showed the loss of dopaminergic neurons in the ipsilateral SN. The dopaminergic neuron terminal loss was also observed in the striatum. There was no obvious discordance observed between the two sides of SN in the vehicle group (Supplementary Figure S1A). The western blot showed similar results. The TH protein level of the nigrostriatal pathway also decreased in the ipsilateral SN in 6-OHDA-lesion rats (Supplementary Figures S1B,C). Collectively, these results proved that a PD unilateral model with stable SNH was successfully established, which is highly consistent with the SNH in PD patients, thus validating the use of this model in subsequent experiments.

### SNH Size Was Positively Related With the Degree of Dopaminergic Neuron Death in PD Rat Model

**FIGURE 2:**
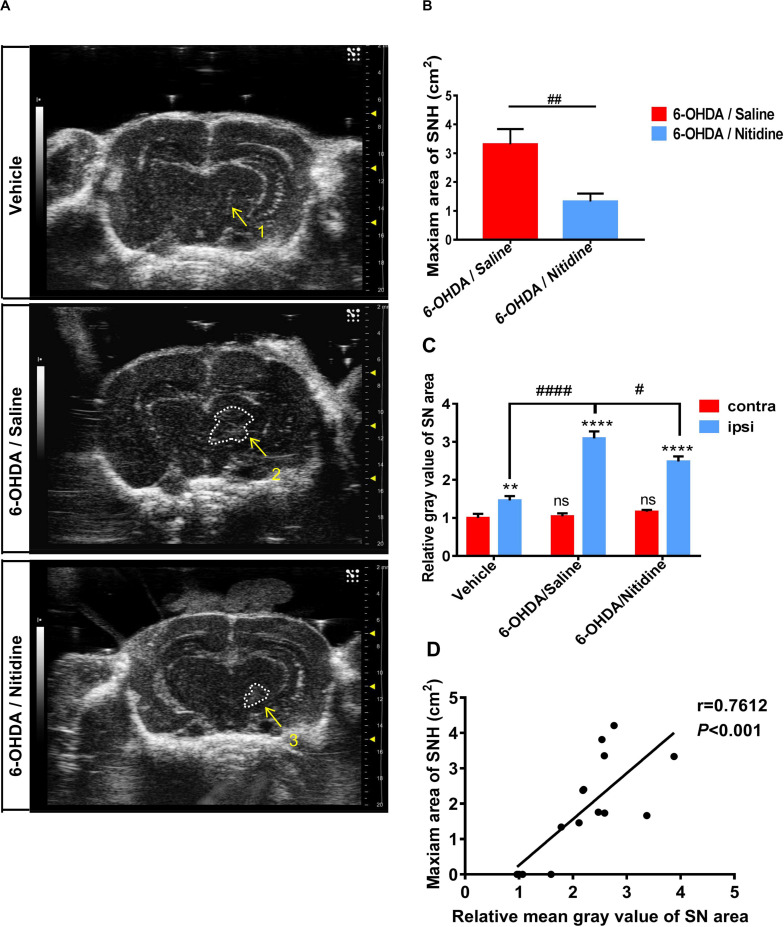
Transcranial sonography (TCS) images and qualification of substantia nigra hyperechogenicity (SNH) after unilateral stereotactic surgery following treatment with nitidine. The rats were pretreated with nitidine (2.5 mg/kg, once daily) or sterile saline for 2 days before surgery and 15 days after surgery. **(A)** The TCS images showed that the SNH area in the midbrain plane of the nitidine-treated group (4, 5) was significantly smaller than that in the saline-treated group (2, 3), while only a scatter echo matching the shape of the needle emerged in the vehicle group (1). Area 2 = 4.211 cm^2^, area 3 = 3.336 cm^2^. **(B)** Statistical result of the maximum SNH areas in different groups (*t*-test). *P* = 0.0069, *t* = 3.776, df = 7, *F* = 1.821. **(C)** Statistical results of the mean gray values of substantia nigra (SN) area. Vehicle group: *t*-test. *P* = 0.0052, *t* = 2.915, df = 52, *F* = 1.086. 6-Hydroxydopamine (6-OHDA)/saline group: *t*-test. *P* < 0.0001, *t* = 10.25, df = 52, *F* = 5.769. 6-OHDA/nitidine: *t*-test. *P* < 0.0001, *t* = 11.25, df = 130, *F* = 7.791. Comparison between contralateral sides: one-way ANOVA. *P* = 0.2051, *F* = 1.606. Comparison between ipsilateral sides: one-way ANOVA. *P* < 0.0001, *F* = 11.43. **(D)** Correlation analysis of mean gray values of SN areas and the maximum SNH area. *P* = 0.0010, *r* = 0.7612, *R*^2^ = 0.5794, *F* = 17.9. *Comparison between contralateral and ipsilateral from a single group. ^#^Comparison between different groups. ns *P* > 0.05, ^#^*P* < 0.05, ^####^*P* < 0.0001, **P < 0.01, ***P < 0.001, ****P < 0.0001.

To further explore whether the size of SNH was positively related with the degree of dopaminergic neuron death in PD models, we applied nitidine, a pentacyclic alkaloid isolated from traditional herbal medicine *Z. nitidium* (Roxb.) DC (Yuan et al., 2015), which shows a considerable neuroprotective effect in PD models, to improve the dopaminergic survival in this PD model (Wang et al., 2016). We found that the SNH areas of nitidine-treated rats were much smaller compared with those of saline-treated ones under TCS (Figures 2A,B). Furthermore, the mean gray value of the ipsilateral SN areas of nitidine-treated rats was reduced compared with the counterparts of saline-treated rats (Figure 2C). A correlation analysis showed that, as per the qualification standards applied to process the TCS images, the SNH areas and the mean gray values of SN were positively correlated (Figure 2D).

**FIGURE 3:**
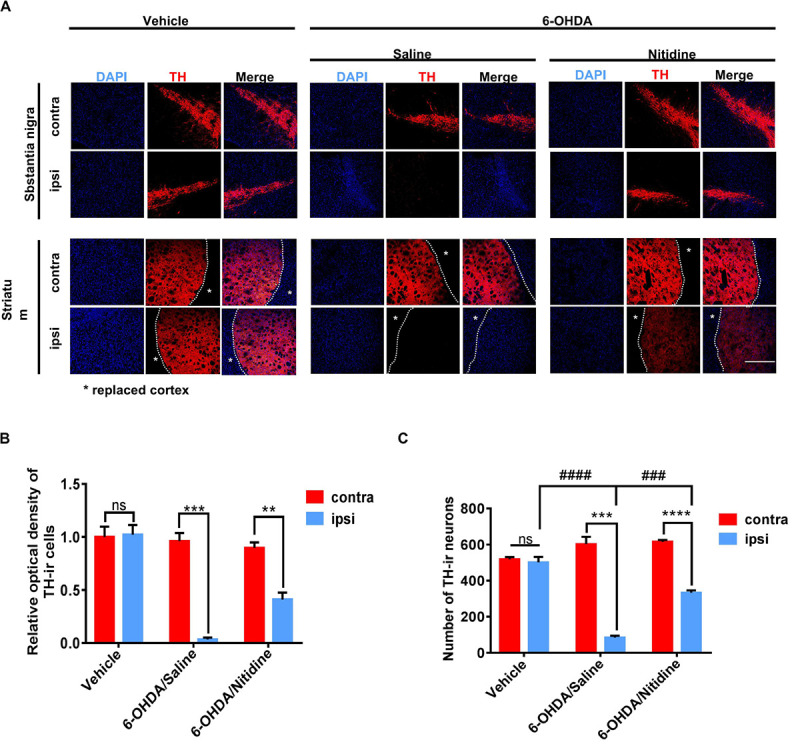
Dopaminergic neuron survival in Parkinson’s disease (PD) models following treatments. **(A)** Tyrosine hydroxylase (TH)-immunoreactive neurons (red) in the PD rats. Scale bar depicts 500 μm. **(B)** Statistical result of optical density values of TH-ir cells in SN (*t*-test). Vehicle group: *P* = 0.8816, *t* = 0.1587, df = 4, *F* = 1.144. 6-Hydroxydopamine (6-OHDA)/saline: *P* = 0.0003, *t* = 11.86, df = 4, *F* = 18.13. 6-OHDA/nitidine: *P* = 0.0043, *t* = 5.853, df = 4, *F* = 1.571. **(C)** Statistical result of TH-ir cell numbers in SN (*t*-test). Vehicle group: *P* = 0.6134, *t* = 0.5472, df = 4, *F* = 6.356. 6-OHDA/saline: *P* = 0.0002, *t* = 12.47 df = 4, *F* = 14.23. 6-OHDA/nitidine: *P* < 0.0001, *t* = 17.85, df = 4, *F* = 1.652. Comparison between ipsilateral sides: one-way ANOVA. *P* < 0.0001, *F* = 113.8. *Comparison between contralateral and ipsilateral from a single group. ^#^Comparison between different groups. ns *P* > 0.05, ^#^*P* < 0.05, ^####^*P* < 0.0001, **P < 0.01, ***P < 0.001, ****P < 0.0001.

Dopaminergic neuron survival was shown by immunofluorescence. It was significantly increased in nitidine-treated rats compared with that in saline-treated ones (Figure 3A). The TH-ir neuron numbers and optical density (OD) values were applied as digitization standards to analyze immunofluorescence results. The histograms showed that the number and the OD values of TH-ir neurons were markedly decreased in the ipsilateral SN compared with those of the contralateral side after 6-OHDA lesion, but the level of reduction in the saline-treated group was significantly higher than that of the nitidine-treated group (Figures 3B,C). Immunohistochemical staining showed similar results (Supplementary Figure S2A). Western blot revealed a remarkable decrease of TH protein level of ipsilateral SN after 6-OHDA lesion, and a moderate increase was observed in the nitidine-treated group but not in the saline-treated ones.

**FIGURE 4:**
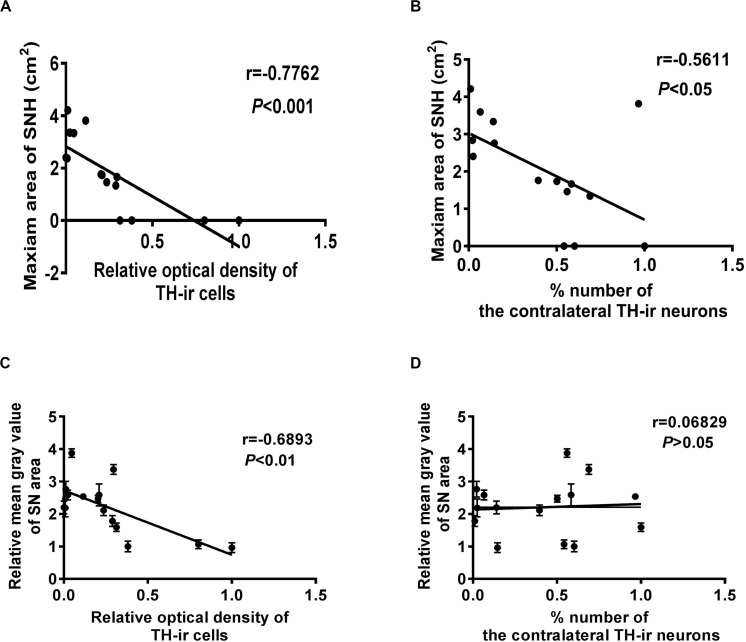
Correlation analysis between different qualification standards of dopaminergic neuron survival and substantia nigra hyperechogenicity (SNH) in Parkinson’s disease (PD). **(A)** Correlation analysis between optical density (OD) values of TH-ir cells and maximum SNH area under transcranial sonography (TCS). *P* = 0.0007, *r* = –0.7762, *R*^2^ = 0.6025, *F* = 19.7. **(B)** Correlation analysis between TH-ir cell numbers and maximum SNH area under TCS. *P* = 0.0296, *r* = –0.5611, *R*^2^ = 0.3148, *F* = 5.972. **(C)** Correlation analysis between OD values of TH-ir cells and mean gray values of substantia nigra (SN) under TCS. *P* = 0.0045, *r* = –0.6893, *R*^2^ = 0.4752, *F* = 11.77. **(D)** Correlation analysis between TH-ir cell numbers and mean gray values of SN under TCS. *P* = 0.8089, *r* = 0.06829, *R*^2^ = 0.004664, *F* = 0.06092.

Correlation statistics was used to analyze the correlation between SNH and dopaminergic neuron survival. It was shown that the TH-ir dopaminergic neuron numbers and the OD values were both negatively correlated with SNH area (Figures 4A,B). The OD values were also negatively correlated with the mean gray values of SN, while the neuron numbers did not show any correlation with it (Figures 4C,D). These results revealed that SNH size was positively related with the degree of dopaminergic neuron death in PD rat model.

### Microglia Activation Might Contribute to SNH Formation

**FIGURE 5:**
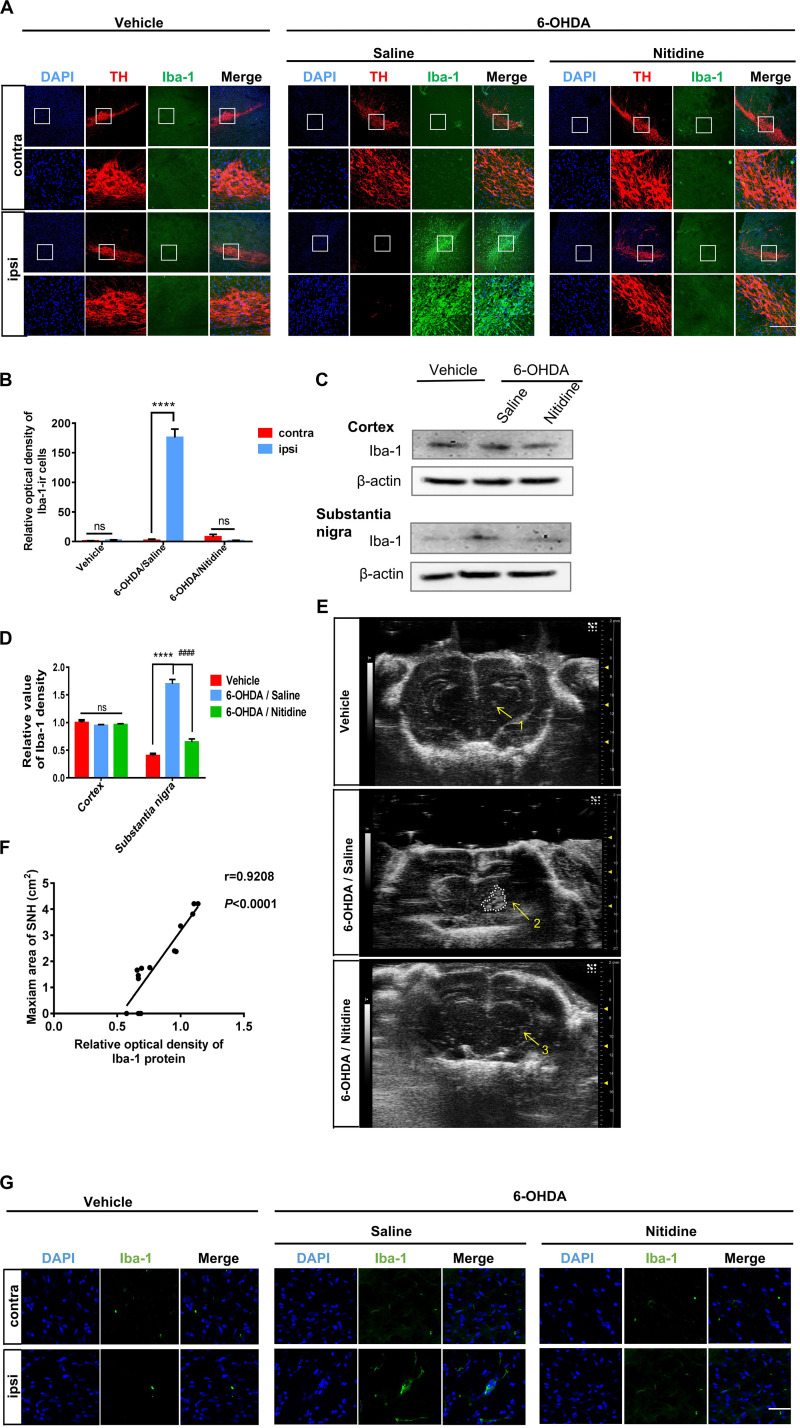
Reactive microgliosis in the substantia nigra (SN) of 6-hydroxydopamine (6-OHDA)-induced Parkinson’s disease rats with or without nitidine treatment. **(A)** Double immunofluorescence of the activated microglia marker Iba-1 (green) and tyrosine hydroxylase-immunoreactive dopaminergic neurons (red). Scale bar depicts 500 μm. **(B)** Statistical result of the optical density values of Iba-1-ir cells. Vehicle group: *t*-test. *P* = 0.0589, *t* = 2.618 df = 4, *F* = 4.355. 6-OHDA/saline group: *t*-test. *P* < 0.0001, *t* = 20.99, df = 4, *F* = 52.36. 6-OHDA/nitidine: *t*-test. *P* = 0.0554, *t* = 2.678, df = 4, *F* = 86.41. **(C)** Iba-1 protein expression revealed by Western blot. **(D)** Histogram showing that the level of Iba-1 protein was significantly decreased in the SN of the nitidine-treated group. One-way ANOVA. Cortex: *P* = 0.4348, *F* = 0.96. Substantia nigra: *P* < 0.0001, *F* = 141.8. **(E)** Transcranial sonography (TCS) images and substantia nigra hyperechogenicity (SNH) qualification after nitidine or saline treatment. Only a scatter echo matching the shape of the needle emerged in the vehicle group (1); the nitidine-treated group did not show visible SNH (3), while the saline-treated group showed a significant SNH (2). Area 2 = 1.111 cm^2^. **(F)** Correlation analysis between Iba-1 protein expression and maximum SNH area under TCS. *P* < 0.0001, *r* = 0.9208, *R*^2^ = 0.848, *F* = 66.93. **(G)** Morphological changes of Iba-1-ir microglia under × 80 lens. Scale bar depicts 3 cm. *Comparison between contralateral and ipsilateral from a single group. ^#^Comparison between different groups. ns *P* > 0.05, ^#^*P* < 0.05, ^####^*P* < 0.0001, **P < 0.01, ***P < 0.001, ****P < 0.0001.

Since the formation mechanism of SNH is still unclear, we aimed to explore the underlying mechanism of hyperechogenicity in the SN area. As microglia are the main cells that take up liberated iron and accumulate iron partly in SN (Mahad et al., 2015), it has been reported to play a critical role in dopaminergic neuron degeneration both in PD patients and 6-OHDA-induced PD models (Swanson et al., 2019). Therefore, we explored whether microglia could be involved in typical SNH in PD models. Immunofluorescence was utilized to observe microglial activation in SN of PD model rats with saline or nitidine treatment. It was shown that a significant increase in microglial activation is marked by ionized calcium binding adaptor molecule-1 (Iba-1) (Rodriguez-Pallares et al., 2007), accompanied by a reduction in TH-ir neurons in PD model rats with saline treatment, while the vehicle group did not present any visible difference between the ipsilateral and the contralateral sides. The microglia activation significantly decreased after nitidine treatment (Figures 5A,B). Western blot displayed similar results. The protein level of Iba-1 in the SN areas of PD model rats was significantly higher than that in the vehicle group rats, while the nitidine-treated group presented a remarkable reduction (Figures 5C,D). In addition, the SNH area under TCS also significantly decreased compared with that in the saline-treated group (Figure 5E). A correlation analysis showed that the Iba-1 protein level was positively correlated with the maximum area of SNH (Figure 5F). Morphological changes of microglia in the ipsilateral SN were observed after nitidine treatment at higher magnification, suggesting that microglia activation was restrained (Figure 5G). Our result showed that microglial activation appeared in the ipsilateral SN area of PD model rats and could be restrained by the neuroprotective drug nitidine. Taken together, our results demonstrated that microglia activation might contribute to SNH formation.

## Discussion

In the present study with 6-OHDA-induced PD rat models, SNH was stably observed through TCS. Importantly, we demonstrated that the size of SNH was positively related with the degree of dopaminergic neuron death in PD models. Finally, we showed that microglia activation might contribute to SNH formation.

Substantia nigra appears as a patchy echogenic structure in the anterior midbrain. According to consensus guidelines (Walter et al., 2007), the hyperechogenicity of SN is defined in terms of an increased size of planimetrically measured SN echogenic area exceeding a pre-defined cutoff value obtained in a normal population. Previous findings proposed an optimal cutoff of 0.24 cm^2^ for differentiating normal controls from patients with enlarged SN (van de Loo et al., 2010) as Berg et al. (2001) reported that SN echogenicity sizes between 0.20 and 0.25 cm^2^ were classified as moderately enlarged, and 0.25 cm^2^ represented the limit for a markedly enlarged SN.

Our results confirmed that the 6-OHDA-induced PD rat model can be used for SNH-related studies. We also found that the appearance of a typical SNH could only be observed 1 week after surgery, noting the crucial value of detecting SNH by TCS for diagnosing PD in the early stage. In addition, recent studies revealed that SNH areas are also correlated with the risk scores and the motor signs, the prediagnostic features of PD (Noyce et al., 2018), indicating its value in preclinical diagnosis.

The result showed that the size of SNH had an inner relationship with the pathological process of PD. However, Berg and colleagues reported that the SNH size was unrelated with disease progression and the gradual neuron loss in SN. They observed PD patients who were assessed by UPDRS I-III. Despite assessments indicating a significant worsening in terms of motor performance, SNH showed little changes. This conclusion should be taken into consideration because 60–70% dopaminergic neurons in SN were lost before clinical symptoms appeared (Postuma et al., 2010), so the measurement which simply chose patients in advanced stages could not offer a time-based detail of how SNH evolved following dopaminergic neuron loss; more extended studies are required. Compared with previous studies, we built PD models with order-of-magnitude dopaminergic neuron loss and explored the relationship between SNH and dopaminergic neuron loss more thoroughly.

Researchers have been interested in looking for suitable markers to assess the process of disease and therapeutic efficacy as the lack of such limited the therapeutic intervention of PD, which had little success. Manganese (Mn)-enhanced magnetic resonance imaging was applied as an imaging biomarker to follow the neurodegeneration of PD (Olson et al., 2016). However, the toxicity of Mn prevented it from being applied to human (Olanow, 2004; Kwakye et al., 2015). Our result offered a new non-invasive and non-toxic method TCS to evaluate the dopaminergic neuron survival or the neuron degeneration in SN of PD. SNH could be utilized as a reliable imaging modality for PD diagnosis and progression monitoring. Moreover, because 60–70% of dopaminergic neurons in SN were lost before symptoms occurred (Postuma et al., 2010; Radhakrishnan and Goyal, 2018), using TCS to directly assess dopaminergic neuron degeneration in SN could evaluate the progression of PD before traditional clinical diagnosis, making it possible for early intervention to be effected during the PD development. Also, SNH is a promising tool to assess the therapeutic effectiveness of PD treatments.

In 6-OHDA-induced PD rat model, we observed SNH with significant microglial activation in the ipsilateral SN. According to several previous studies, (1) SNH reflects pathological alterations related to an increased SN iron content (Berg et al., 1999, 2002; Zecca et al., 2006); (2) liberated iron is then taken up mainly by microglia and macrophages and accumulates partly in these cells (Mahad et al., 2015), and (3) activated microglia are involved in the deficiency and degeneration of dopaminergic neurons in the SN (Langston et al., 1999; Marinova-Mutafchieva et al., 2009; Xu et al., 2016) and can be observed in postmortem studies (Mcgeer et al., 1988; Yuan et al., 2015). We drew a conclusion that SNH microglia activation might contribute to the formation of SNH. Our results also hinted that inhibiting the activation of microglia may be the future direction for the prevention and the treatment of PD.

Our research also had some limitations. First, animal PD models like 6-OHDA rat model and 1-methyl-4-phenyl-1,2,3,6-tetrahydropyridine mouse model cannot fully simulate the whole pathological process of human PD. A previous study especially showed that neuromelanin played an important role in PD pathogenesis, and its accumulation might tend to reduce SNH (Zecca et al., 2008; Berg et al., 2010), while neuromelanin was absent in rodents and many other species (Marsden, 1961). Notably, increasing studies confirmed that extracellular neuromelanin leads to microglial activation (Langston et al., 1999; Zecca et al., 2008; Ferrari et al., 2016). Moreover, after correcting the neuromelanin contents in the SN of PD patients, microglia activation was still related with SNH (Berg et al., 2010). Our current study showed that microglia activation contributed to SNH formation in the 6-OHDA rat midbrain. Taken together, we speculate that microglia activation is the shared and the convergent key point for SNH formation in either PD animal models or human PD. However, further studies are warranted to prove this speculation.

## Data Availability Statement

All datasets presented in this study are included in the article/Supplementary Material.

## Ethics Statement

The animal study was reviewed and approved by Institutional Animal Care and Use Committee (IACUC-20190603).

## Author Contributions

SZ designed and performed the experiments. KT helped performed the experiments and contributed to reag ents and analysis tools. JW provided ultrasonography process. YD helped perform the analysis with cons tructive discussions. XL is the corresponding author. All authors contributed to the article and approved the submitted version.

## Conflict of Interest

The authors declare that the research was conducted in the absence of any commercial or financial relationships that could be construed as a potential conflict of interest.

## Supplementary Material

FIGURE S1 Evaluation of stereotactic 6-hydroxydopamine (6-OHDA) injection in the Parkinson’s disease rat model. **(A)** Dopaminergic neurons were stained for immunofluorescence by tyrosine hydroxylase (TH) immunoreactivity. Scale bar depicts 300 μm. **(B)** TH protein levels in the SN and the striatum of 6-OHDA-lesioned and vehicle rats. **(C)** The ratios of TH to total protein loading (β-actin) are shown in the histogram (*t*-test). Striatum of the vehicle group: *P* = 0.1911, *t* = 1.572, df = 4, *F* = 1.384. Striatum of the 6-OHDA group: *P* < 0.0001, *t* = 46.68, df = 4, *F* = 1.385. Substantia nigra of the vehicle group: *P* = 0.4934, *t* = 0.7529, df = 4, *F* = 1.439. Substantia nigra of the 6-OHDA group: *P* < 0.0001, *t* = 22.86, df = 4, *F* = 32.66. ^****^*P* < 0.0001. ^#^Comparison between different groups. ns *P* > 0.05, ^#^*P* < 0.05, ^####^*P* < 0.0001, ***P* < 0.01, ***P* < 0.01, ****P* < 0.001, *****P* < 0.0001.

FIGURE S2 **(A)** Immunochemistry staining with tyrosine hydroxylase (TH) antibody showing TH-immunoreactive cells. **(B)** Western blot showing the TH protein level. **(C)** Statistical result of the TH protein expression levels. One-way ANOVA. Cortex: *P* = 0.3580, *F* = 1.225. Striatum: *P* < 0.0001, *F* = 593.3. Substantia nigra: *P* < 0.0001, *F* = 6452. ^#^Comparison between different groups. ns *P* > 0.05, ^#^*P* < 0.05, ^####^*P* < 0.0001, ***P* < 0.01, ****P* < 0.001, *****P* < 0.0001.
