# Supplementary figures and images for: Substantia Nigra Hyperechogenicity Reflects the Progression of Dopaminergic Neurodegeneration in 6-OHDA Rat Model of Parkinson’s Disease
Source: Front Cell Neurosci. 2020 Aug 4;14:216. doi: 10.3389/fncel.2020.00216 (PMC7418516; doi:10.3389/fncel.2020.00216)

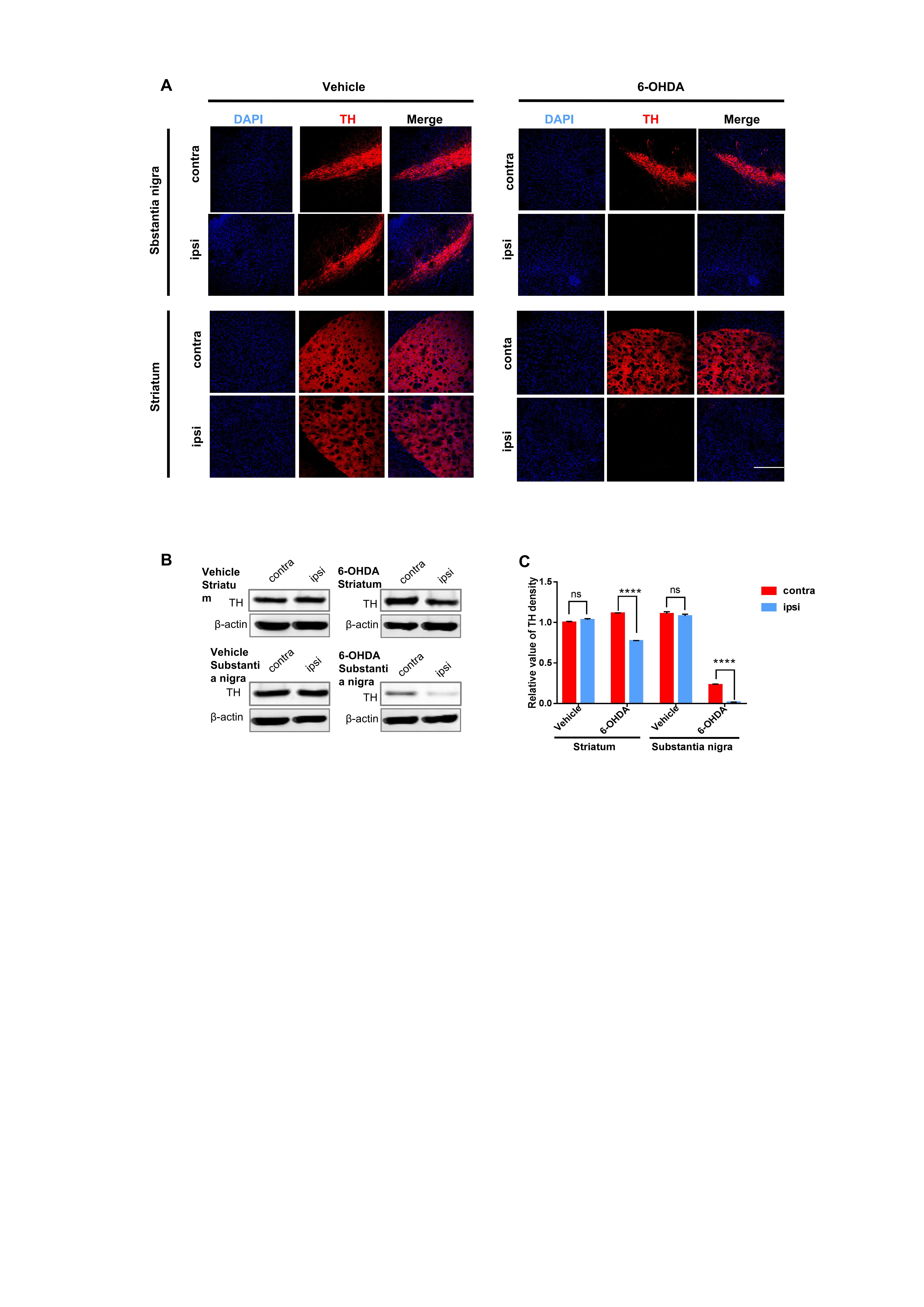

Supplement: FIGURE S1 — Evaluation of stereotactic 6-hydroxydopamine (6-OHDA) injection in the Parkinson’s disease rat model. (A) Dopaminergic neurons were stained for immunofluorescence by tyrosine hydroxylase (TH) immunoreactivity. Scale bar depicts 300 μm. (B) TH protein levels in the SN and the striatum of 6-OHDA-lesioned and vehicle rats. (C) The ratios of TH to total protein loading (β-actin) are shown in the histogram (t-test). Striatum of the vehicle group: P = 0.1911, t = 1.572, df = 4, F = 1.384. Striatum of the 6-OHDA group: P < 0.0001, t = 46.68, df = 4, F = 1.385. Substantia nigra of the vehicle group: P = 0.4934, t = 0.7529, df = 4, F = 1.439. Substantia nigra of the 6-OHDA group: P < 0.0001, t = 22.86, df = 4, F = 32.66. ****P < 0.0001. #Comparison between different groups. ns P > 0.05, #P < 0.05, ####P < 0.0001, **P < 0.01, **P < 0.01, ***P < 0.001, ****P < 0.0001. [file Image_1.JPEG]

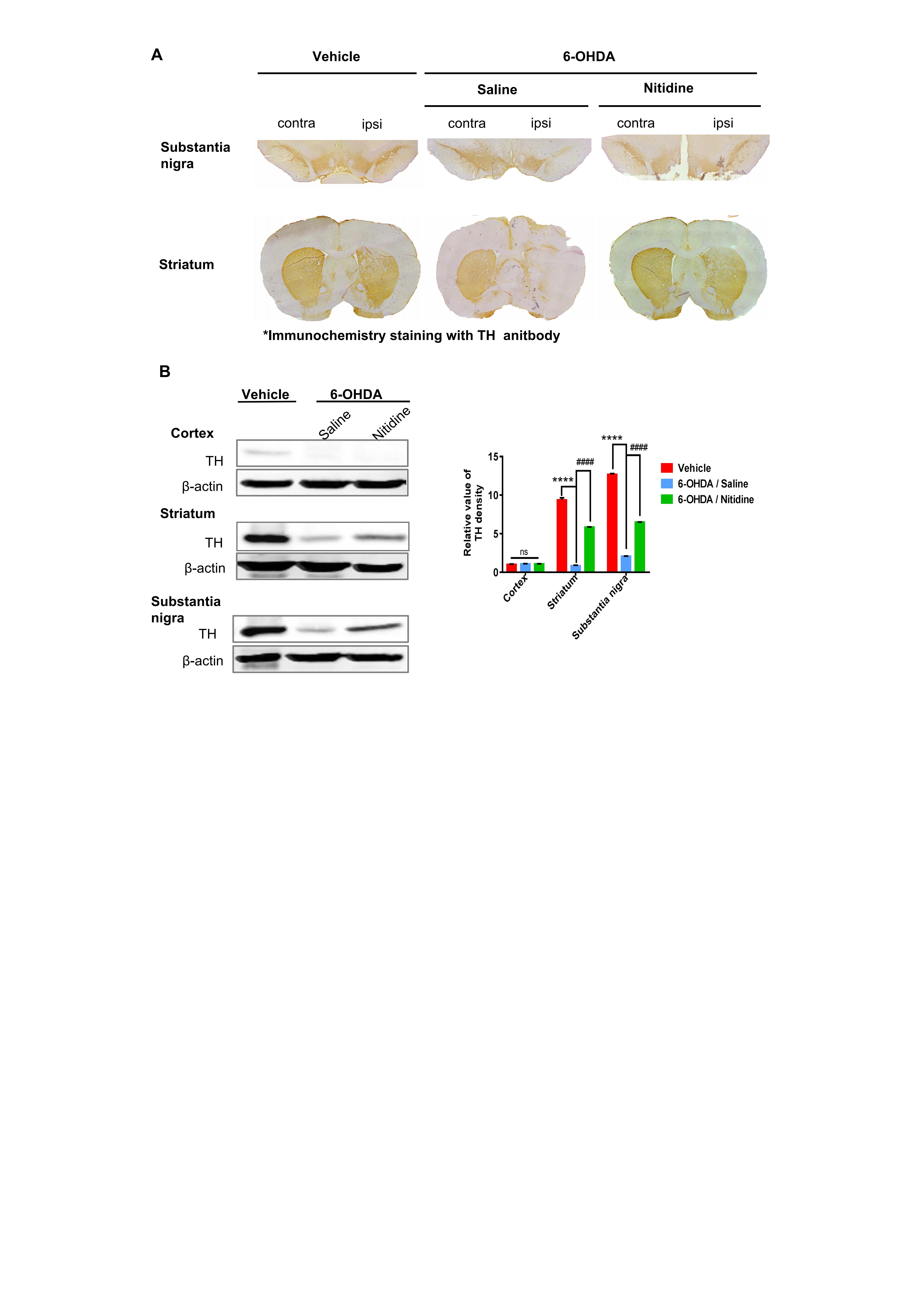

Supplement: FIGURE S2 — (A) Immunochemistry staining with tyrosine hydroxylase (TH) antibody showing TH-immunoreactive cells. (B) Western blot showing the TH protein level. (C) Statistical result of the TH protein expression levels. One-way ANOVA. Cortex: P = 0.3580, F = 1.225. Striatum: P < 0.0001, F = 593.3. Substantia nigra: P < 0.0001, F = 6452. #Comparison between different groups. ns P > 0.05, #P < 0.05, ####P < 0.0001, **P < 0.01, ***P < 0.001, ****P < 0.0001. [file Image_2.JPEG]

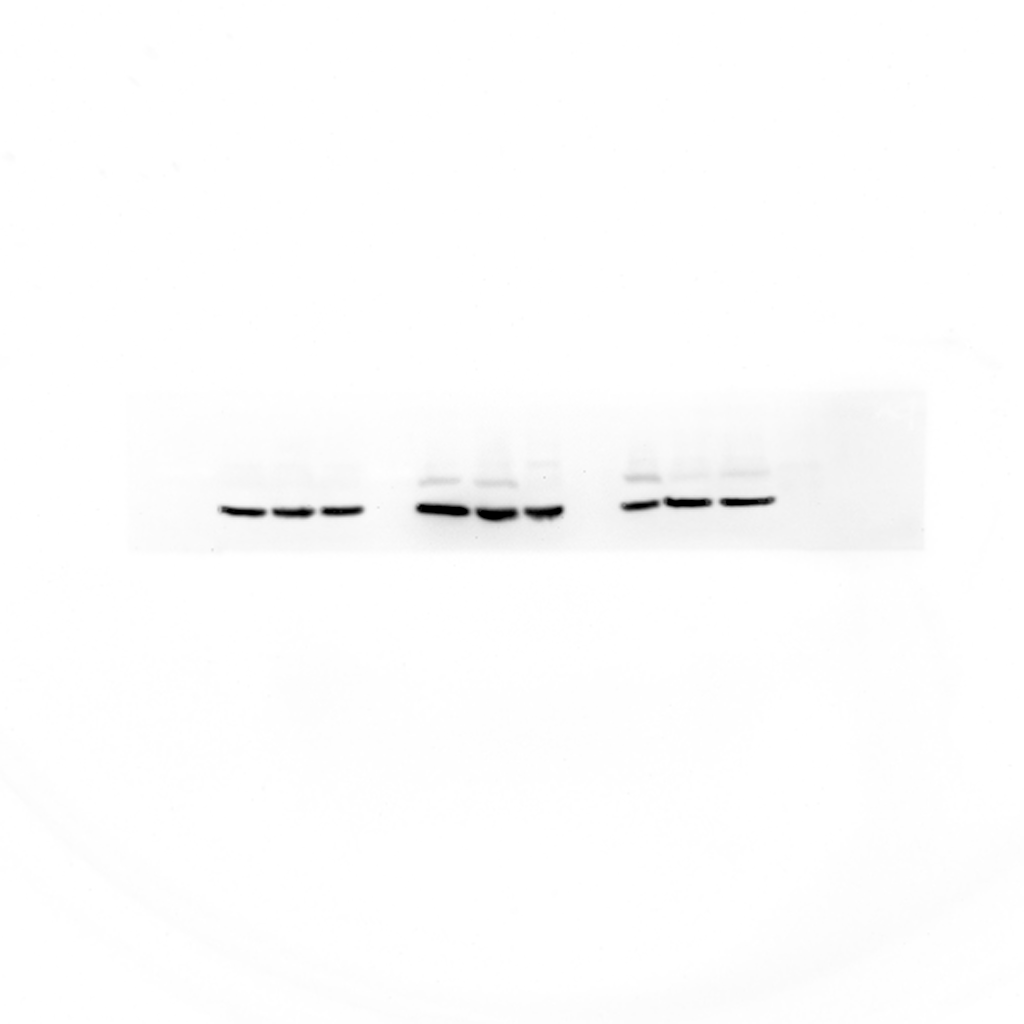

Supplement: Supplementary file 3 [file Image_3.TIF]

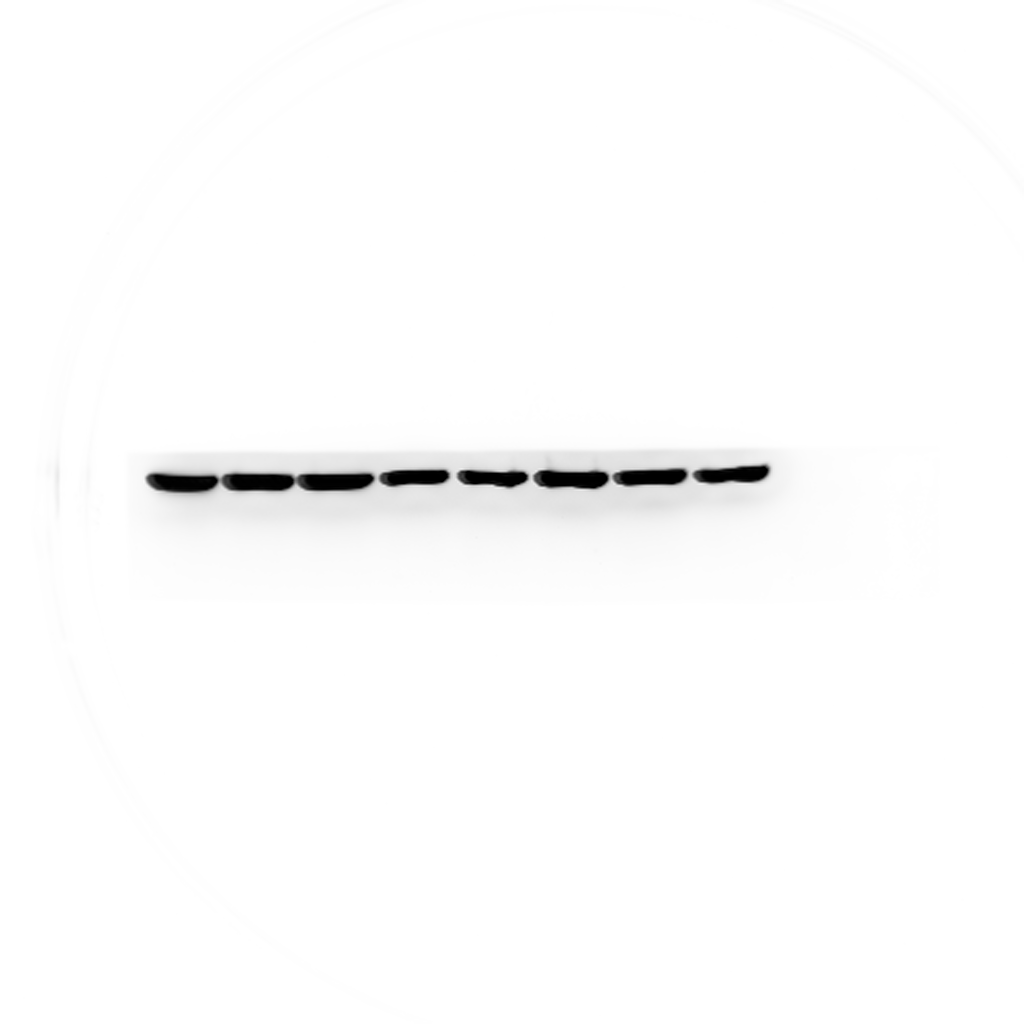

Supplement: Supplementary file 4 [file Image_4.TIF]

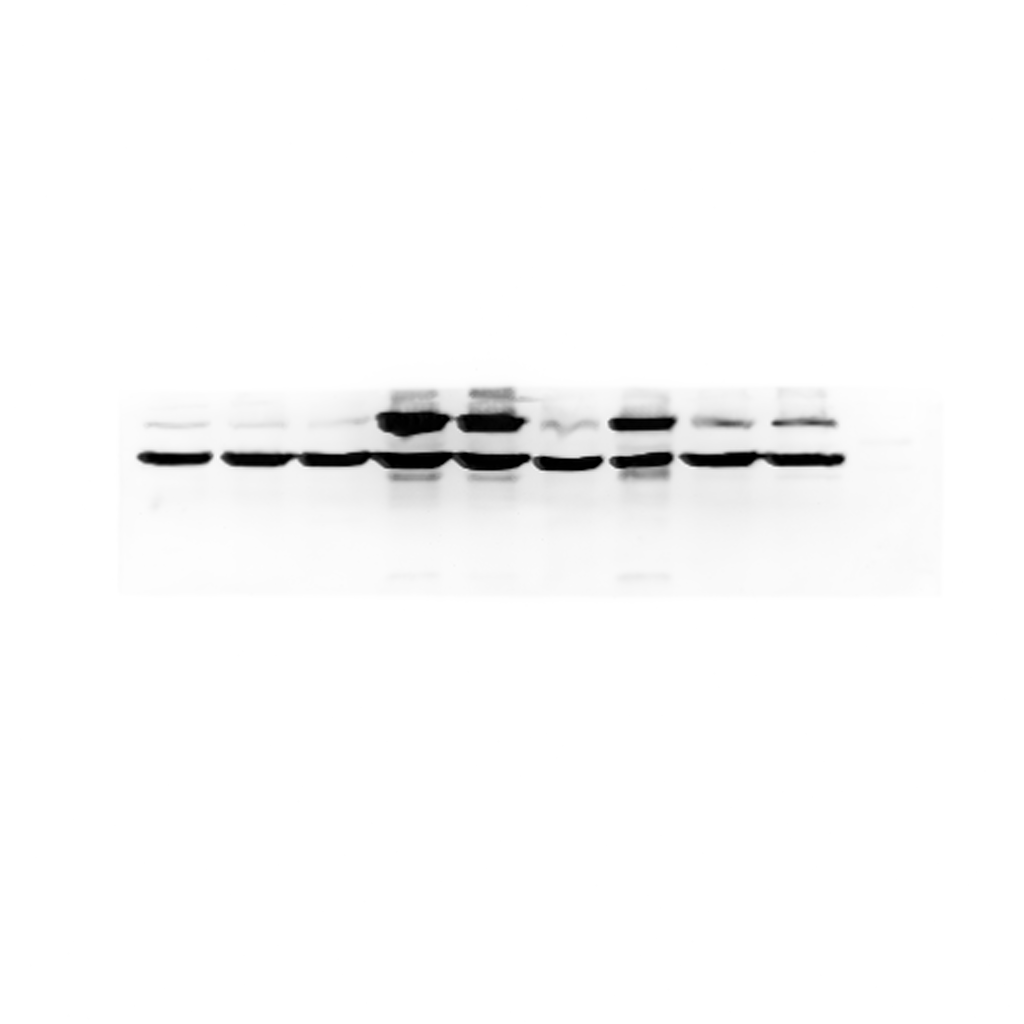

Supplement: Supplementary file 5 [file Image_5.TIF]

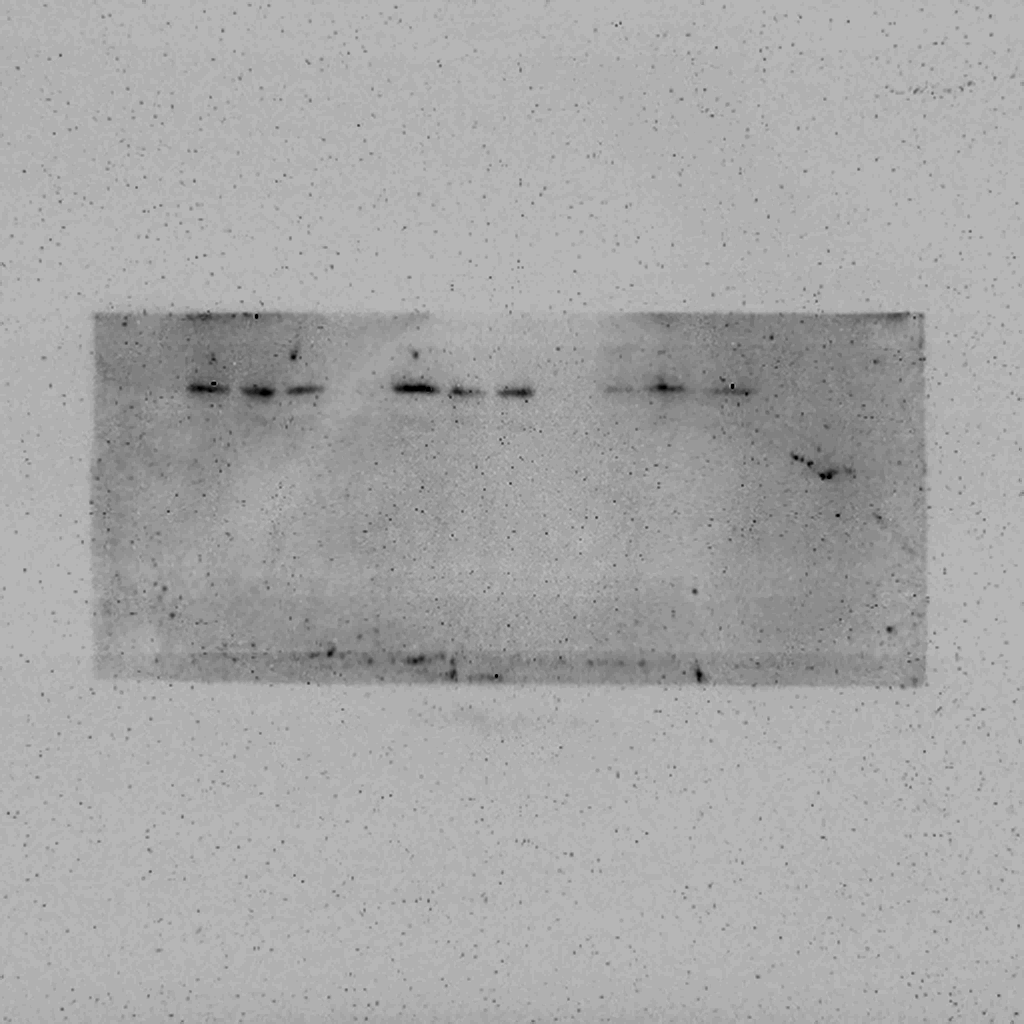

Supplement: Supplementary file 6 [file Image_6.TIF]

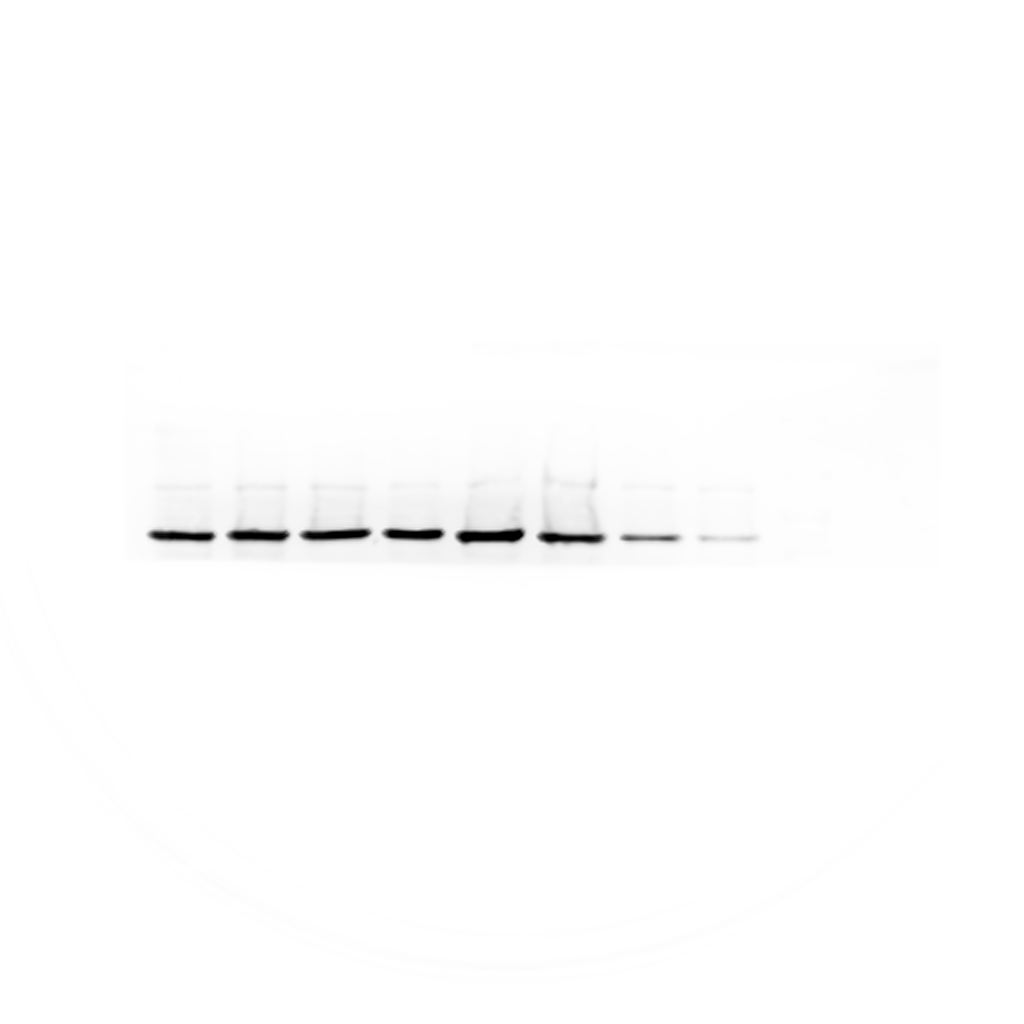

Supplement: Supplementary file 7 [file Image_7.TIF]

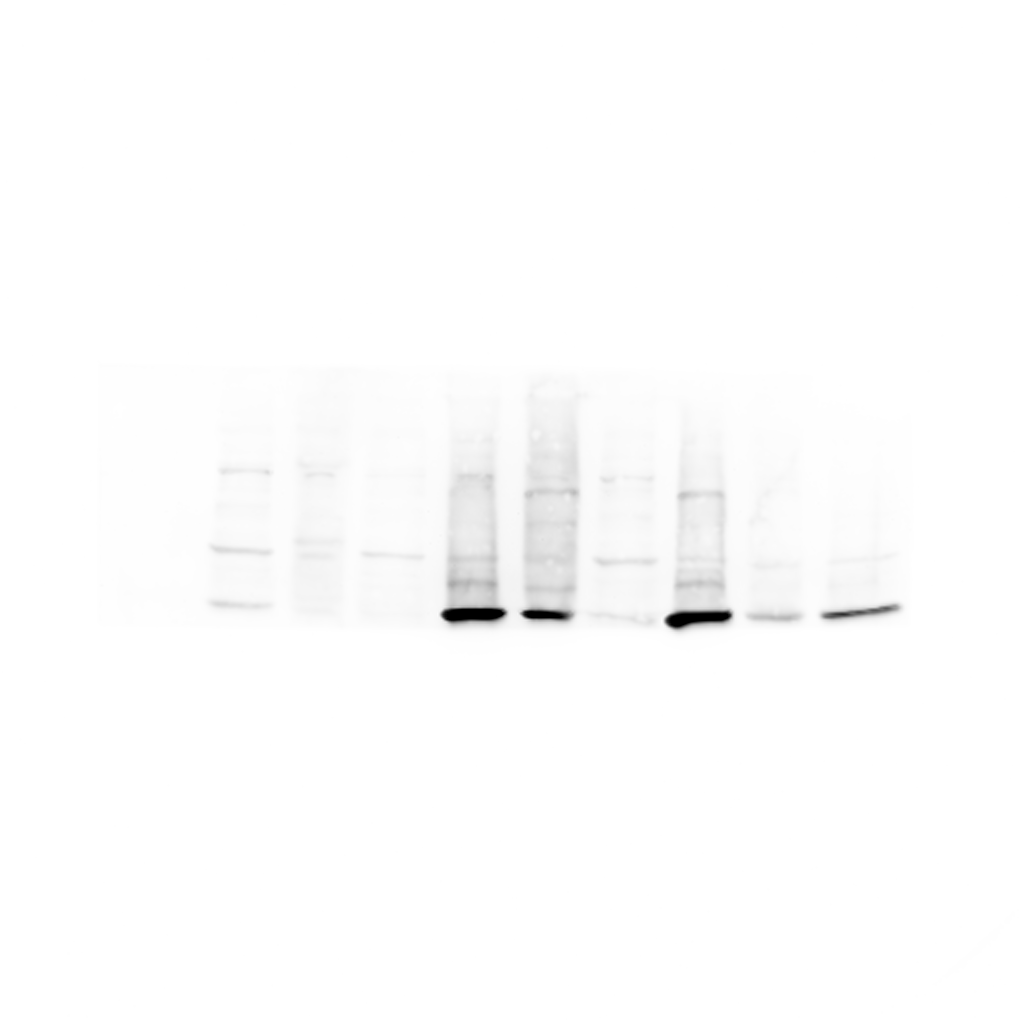

Supplement: Supplementary file 8 [file Image_8.TIF]
